# Composition and Laboratory Correlation of Commercial Probiotics in India

**DOI:** 10.7759/cureus.11334

**Published:** 2020-11-05

**Authors:** Dhanasekhar Kesavelu, Anusha Rohit, Iddya Karunasagar, Indrani Karunasagar

**Affiliations:** 1 Pediatric Gastroenterology, Apollo Children’s Hospital, Chennai, IND; 2 Pediatrics and Child Health, SS Child Care, Chennai, IND; 3 Microbiology, Madras Medical Mission, Chennai, IND; 4 Microbiology, NITTE University, Mangalore, IND

**Keywords:** probiotics, commercial, targeted metagenomics, ngs, india, quality, paediatrics, safety, cell count, 16srna

## Abstract

Objectives

Probiotics are defined as live microorganisms that, when administered in adequate amounts, confer health benefits to the host. Probiotics are currently being recommended and considered for many medical conditions. The Asia-Pacific region contributes to more than 40% of the global industry. Quality of commercial probiotics remains a challenge globally and has been a major concern in various countries in Europe, South Africa, Taiwan, India, Pakistan, and the USA. Research from these countries indicate that the contents do not correspond to the label information in terms of identity, viability, number of microorganisms or purity. The objective of this study is to assess the commercial probiotic bacterial contents and their label accuracy in India. No previous research has been done in this area in India, on commercial probiotics that are sold as “pharmaceuticals”.

Methods

A random selection of the most prescribed probiotics for various clinical indications were chosen with a minimum shelf life of 12 months. The probiotics were single and multiple strains and these were evaluated by culture, viable plate count, DNA isolation and targeted metagenomics. Our study is the first step in scrutinizing probiotics in terms of quality and quantity analysis which are used across various age groups for multiple indications.

Results

Out of the 20 chosen probiotics eight products were single strain and 12 products were multiple strains. These probiotics showed very poor correlation between the declared contents on the pack and lab values in viable cell count colonies, the genus and species strain identification, presence of contaminants and these were confirmed with 16s RNA and next generation sequencing.

Conclusion

Poor correlation in the quality and quantity of probiotics proves that the label claim and actual claim of these “drugs” show exceptionally poor correlation and raises safety concerns in clinical use, especially in vulnerable age groups such as neonates, children and the elderly. Our study shows that “policing” of these probiotics is essential in protecting these patients who are at risk and ensuring quality control and helping clinicians making the right choice.

## Introduction

Critical analysis of medical probiotics

According to the International Scientific Association for Probiotics and Prebiotics, probiotics are defined as “live microorganisms that, when administered in adequate amounts, confer a health benefit on the host” [1]. Probiotics have the potential to bridge the pharmacological and non-pharmacological fields in the treatment of a variety of diseases and disorders. The probiotic market has been growing rapidly; the global probiotic market was valued at 36.6 billion USD in 2015 and is expected to grow at a 7% compound annual growth rate (CAGR) from 2016 to 2023, with Asia-Pacific currently contributing >40% of the global share [2]. India, China, and Japan are major global stakeholders in the probiotic market, and India and China will likely see colossal growth in the next decade.

Growing awareness in health, lifestyle, and increasing issues related to metabolic and digestive disorders is a major contributing factor for the precipitous increase in probiotic market shares [3,4]. Various medical bodies and organizations such as the European Society of Paediatric Gastroenterology, Hepatology, and Nutrition [5] and the World Gastroenterology Organization [6] have produced guidelines and recommendations for probiotics in the treatment of multiple diseases and disorders with varying levels of evidence for each disease/condition. The significant factors for choosing a probiotic include genus and species identification, strain designation, viable count of each strain at the end of shelf-life, recommended storage conditions, safety, recommended dose, an appropriate description of the physiological effect, and contact information for post-market surveillance [6].

Multiple studies exist internationally concerning the quality of commercial probiotics; European countries [7,8], the USA [9], and South Africa [10,11] retain their autonomous regulatory bodies, which have produced guidelines and recommendations on probiotic use [12-14].

There are no standards to check the quality of commercial probiotics routinely in India; however, the Indian Council of Medical Research issued guidelines on the quality of probiotics in 2015 [15]. The Indian probiotic market is flooded with myriad probiotics that confuse health care professionals. The most recent edition of the Indian drug formulary (i.e., the Current Index of Medical Specialities) lists over 160 probiotic brands available in India with various single strains and multiple combinations of various strains. Commercial probiotics are available in different forms (e.g., dry powder sachets, capsules, liquid formulations, dry powder syrup, and in combination with antibiotics), and some manufacturers claim its existence in infant formulae and oral rehydration salts.

The quality analysis of commercial probiotics has been conducted using a variety of techniques worldwide. Over the past decade, bacterial/spore counts and genetic tests have evaluated bacterial species and strains in probiotics licensed for medical indications. Recently, there has been a profound change in the way the species and strains of probiotics are identified [16]. Terminal restriction fragment length polymorphism analysis can determine the bacterial composition of probiotics [9], and deoxyribonucleic acid (DNA) extraction and polymerase chain reaction (PCR) are useful in verifying label claims [9]. Research using single conventional and/or molecular techniques have revealed significant discrepancies between actual and label claims of commercial probiotics [11,17,18]. We conducted this study to verify the actual content compared to label claims of commercial probiotics in India using a combination of a conventional culture method screening and DNA isolation and targeted metagenomic analysis.

## Materials and methods

Sample and culture technique

A total of 20 probiotics (nine single strain probiotics and 11 multiple strain probiotics) were bought from the local pharmacy (over the counter) with an expiration date a minimum of 12 months from the date of purchase. We tested the contents initially by using conventional culture techniques at Chennai. Our local hospital ethics committee approved the study design before the start of the study. Each probiotic was tested in three different batch numbers and with a minimum expiry date of 12 months.

Depending on the contents declared on the sachet, 1 g of the probiotic powder was first inoculated onto 5 ml of liquid broth, such as de Man, Rogosa, and Sharpe broth (MRS broth; Sigma Aldrich, India), yeast extract peptone broth (MicroExpress, Tulip Diagnostics, Goa, India) and thioglycolate broth (MicroExpress, Tulip Diagnostics, Goa, India).

Serial tenfold dilutions up to 106 were made in phosphate buffer saline and plated on MRS agar or tryptic soy agar (Microexpress, Tulip Diagnostics, Goa, India) using pour plate technique and incubated under aerobic or anaerobic conditions using an anaerobic jar and gas pack (BioMérieux, Marcy l’Etoile, France) as appropriate for the expected organism. After incubation for 24 to 48 hours at 37°C, the colonies were counted. Smears from single colonies were stained with Gram staining, and the isolated organisms were identified using a Vitek® II Compact Anaerobe and Corynebacteria test card (BioMérieux, Marcy l’Etoile, France) or yeast card as appropriate. The cultures were subsequently followed by matrix-assisted laser desorption/ionization-time-of-flight-mass spectrometry [19,20] to identify the species and strain of the probiotics (Table 1).

**Table 1:** Commercial probiotics used for analysis. *Single strain probiotic

PRODUCT NAME	BATCH	QUANTITY RECEIVED	EXPIRY
ECONORM*	3168	4 sachets (0.75 g each)	May 2021
BENEGUT*	VBD0080	2 vials (5 mL each)	June 2020
REMUNE AL	12SRL020	2 sachets (1 g each)	December 2020
ENTEROGERMINA*	11187	4 vials (5 mL each)	December 2020
BIFILAC GG*	AF19006/AF19010	3 sachets (0.75 g each)	June 2020
BIFILAC	LLA9U2	4 sachets (0.5 g each)	November 2020
PRE PRO KID	15SPR132	4 sachets (1 g each)	December 2019
REGUTOL	EP8744003	2 vials (30 mL each)	July 2019
CYFOLAC*	K0118	2 vials (5 mL each)	January 2020
GUT PRO*	XGW8002	2 vials (20 mL each)	June 2019
COMBIFLORA	PSB18SA29	4 sachets (1 g each)	June 2020
GNORM*	NGS 1923	4 sachets (0.765 g each)	July 2020
DAROLAC	M1097F358	3 sachets (1 g each)	November 2019
PRE PRO KID L	FS18002	3 sachets (1 g each)	December 2019
VIBACT	AA18D2	6 capsules	April 2020
VIZYLAC*	8065631-9092	6 capsules (0.3 g each)	January 2020
REFLORA Z	12SR0010	2 sachets (1 g each)	December 2019
SUPER FLORA GG	12SSG015	2 sachets (1 g each)	July 2020
SPORLAC	SPS9B013	8 sachets (1 g each)	June 2020
ENTEROPLUS*	4797	1 sachet (1 g each)	October 2019

Analytical procedure

There are no global standard methods for analyzing commercial probiotics; therefore, we adopted the methodology described by Aureli et al. to determine the number of culturable probiotic cells [21]. These methods are the official reference methods of analyzing probiotic food supplements in Italy. All procedures were performed in a biosafety cabinet to protect samples from contamination. The contents of these capsules, vials, and sachets were collected and mixed, wherever possible, to take a sample of the representative batch. One gram of the sample was weighed, dissolved in 9 ml of Maximum Recovery Diluent (Thermo Fisher Scientific, Waltham, MA), and a 10-fold dilution series was made. For spore-forming bacteria, the first suspension was incubated at 80°C in a water bath for 10 minutes before further dilution to kill vegetative cells and allow germination of spores. The number of probiotic cells was determined by spread plating 100 μL of each dilution on respective selective media according to the label-claimed organisms. Each dilution was plated in duplicate to avoid errors.

Because the culture-based method for quality control of probiotic products does not cover all the different microorganisms present in the commercial products under investigation, additional culture methods suitable for quality control were employed. Official International Organization for Standardization standards for the microbiology of food and animal feeding were employed where available [22]. The quantification of microorganisms in each sample was achieved by counting the total number of colony-forming units (CFU) grown on an agar plate from serial dilutions (Table 2).

To calculate the final CFU, the following formula was used [23]:

N₌∑C/(n1+0,1*n2) *d

Where ∑C is the sum of characteristic colonies counted on all dishes retained, n1 is the number of dishes retained in the first dilution, n2 is the number of dishes retained in the second dilution, and d is the dilution factor corresponding to the first dilution retained. The resulting colonies were multiplied by the dilution factor and averaged between the replicates. Results were expressed as the number of CFU per g content of the capsule sachet or mL of the vial.

**Table 2:** Studies reporting discrepancies between product labeling and independent laboratory analysis of species contained in probiotic products. *Single strain probiotic NGS: Next-generation sequencing.

Commercial probiotic	Claimed contents	Actual contents as per laboratory	NGS
ECONORM*	S. boulardii	S. boulardii	S. boulardii
BENEGUT*	B. clausii	B. clausii	Bacillus thuringiensis (55%), Paenibacillus popilliae (41%)
REMUNE AL	L. paracasei, L. fermentum	None	None
ENTEROGERMINA*	B. clausii	B. clausii	B. clausii (100%)
BIFILAC GG*	L. rhamnosus	L. rhamnosus	Lactobacillus rhamnosus (97%)
BIFILAC	S. faecalis, Lactic Acid bacillus, B. mesentericus, C. butyricum	Enterococcus hirae (65%), Bacillus coagulans (25%), C. butyricum (0.2%)	Enterococcus hirae (65%), Bacillus coagulans (25%), C .butyricum (0.2%)
PRE PRO KID	L. acidophilus, B. longum, B. infantis	L. rhamnosus	Bifidobacterium animalis subsp lactis (70.3%), L.plantarum (15.8%)
REGUTOL	B. subtilis HU058	B. subtilis and B. coagulans	Bacillus subtilis subsp (79.6%), B. sonorensis (15.18%), B. thuringiensis serovar israelensis ATCC 35646 (1.5%)
CYFOLAC*	B. clausii	B. clausii	B. clausii (99.9%) B. clausii ATCC 21636 and KSM-K16 strains
GUT PRO*	B. clausii	B. clausii	B. clausii (99.65%)
COMBIFLORA	L acidophilus, Bifidobacterium longum, Bifidobacterium lactis, Saccharomyces boulardii, Lactic acid bacillus	Bifidobacterium animalis subsp. lactis, B. coagulans	Bifidobacterium animalis subsp. lactis (45%), B. coagulans (50%), L.acidophilus (1.14%)
GNORM*	S. boulardii	S. boulardii	S. boulardii
DAROLAC	Lactobacillus rhamnosus, Lactobacillus acidophilus	B. longum, L. rhamnosus	B. longum (subsp. infantis, 85%), L. rhamnosus (15%), L. acidophilus (0.025%)
PRE PRO KID L	S. boulardii. L. rhamnosus GG	B. animalis subsp., Enterococcus hirae, L. rhamnosus	Bifidobaterium animalis subsp lactis (29.6%), Enterococcus hirae (58.38%), L. rhamnosus (1.97%), L. plantarum, Uncultured bacterium (0.8%)
VIBACT	Streptococcus fecalis, Clostridium, butyricum, Bacillus mesentericus, Lactic acid bacillus	E. hirae, B. coagulans	E. hirae (43.91%), Bacillus sonorensis (36.9%), Clostridium butyricum (0.5%), Uncultured bacterium (17.5%)
VIZYLAC*	Lactic acid bacillus, L. sporogenes	B. coagulans	B. coagulans (99.21%)
REFLORA Z	S. boulardii, Lactic acid bacillus	B. coagulans	B. coagulans (>99%)
SUPER FLORA GG	L. rhamnosus	L. rhamnosus	L. rhamnosus (62.2%), L. rhamnosus (35.35%)
SPORLAC	L. sporogenes	B. coagulans	B. coagulans (>99%)
ENTEROPLUS*	L. rhamnosus	L. rhamnosus	L. rhamnosus (61.4%), L. rhamnosus (36.8%)

DNA isolation

The Fast DNA® SPIN Kit for Soil (MP Biomedicals, Italy) with the Fast Prep®-24 Instrument following the manufacturer’s instructions was used to isolate DNA directly from probiotics products. A total of 200 mg of powder (for sachets and capsules) was used, or pellets collected after centrifugation of 3 ml suspension (for vials) was used. DNA quantity was measured with the Qubit® dsDNA HS Assay Kit and the Qubit® Fluorometer (Life Technologies, Thermo Fisher, Italy), and DNA integrity was verified using 1% agarose gel electrophoresis.

For DNA purification from colonies, a single isolated colony from a pure culture of each selected agar plate was suspended in 20 µL of MicroLYSIS® (Microzone, Clent Life Science, United Kingdom). The suspension was heated in a thermal cycler to extract yeast DNA following manufacturer instructions.

Targeted metagenomic analysis

To conduct targeted metagenomics analysis, we followed the process first published by Patrone et al. as follows [24]. DNA amplifications were carried out using the primers 343F (5′-TACGGRAGGCAGCAG 3′) and 802R (5′-TACNVGGGTWTCTAATCC-3′) targeting the V3-V4 regions of the bacterial 16S ribosomal ribonucleic acid (rRNA) gene. A specific seven-base long tag was attached to forward primer to assign sequences to samples during bioinformatics analysis. For each sample, the PCR amplification was performed in triplicate using 2 ng of DNA for each reaction. The PCR protocol included an initial denaturation (95°C, 3 minutes), followed by 23 cycles of denaturation at 94°C for 30 seconds, annealing at 52°C for 30 seconds, and extension at 72°C for 30 seconds, with a final extension at 72°C for 7 minutes. Each amplification reaction was carried out in a 25-µl mixture with 1 µl DNA, 0.5 μM of each forward and reverse primer, and 1X KAPA® SYBR FAST qPCR Master Mix (KAPA Biosystems, Wilmington, MA). Following amplification, the PCR products were checked by agarose gel electrophoresis and quantified using the Qubit HS® dsDNA fluorescence assay (Life Technologies®, Thermo Fisher, Italy). Amplicons were pooled in equimolar concentration and purified by the Agencourt AMPure® XP PCR1 Purification system (Beckman Coulter, Brea, CA, USA). Sequencing was performed at Fasteris (Geneva, Switzerland) using Illumina’s MiSeq platform with 300 bp paired-end mode and v3 chemistry. After quality control of the raw data using Fast QC v0.11.2 (Babraham Bioinformatics, Cambridge, UK), Trimmomatic v0.32 (USADEL LAB, Aachen, Germany) was used for the quality filtering of raw reads by trimming regions having a quality value lower than 20 (Phred-scale) over a four-base wide sliding window, and to remove reads shorter than of 36 nucleotides. The ea-utils v.1.1.2‐537 fastq-join tool (TRIAD National Security, LLC, Los Alamos, NM) was used to merge overlapping paired-end reads [25]. Assembled sequences were de-replicated, sorted, and clustered into operational taxonomic units (OTUs) at 97% identity using VSEARCH v1.0.14 following standard UPARSE pipeline parameters (UPARSE is a method for generating clusters [OTUs] from next-generation sequencing reads of marker genes such as 16S rRNA, the fungal internal transcribed spacer [ITS] region and the cytochrome oxidase subunit 1 [CO1] gene). Chimeric sequences were detected using the UCHIME algorithm (an algorithm for detecting chimeric sequences) and removed from further analysis. Taxonomy was assigned by aligning these OTU sequences against SILVA 137 reference database using the program NCBI-Blast v2.2.27. OTU-table and taxonomy-table files were created using custom scripts [24].

Confirmation of Saccharomyces cerevisiae subspecies boulardii

Although bacterial probiotic species were distinguished using selective culturing and colony morphology and identified through Illumina sequencing, Saccharomyces cerevisiae subspecies (subsp.) boulardii was not obviously checked by 16S rDNA amplicon sequencing. The identity of cultures isolated on Yeast Peptone broth medium was thus confirmed by PCR amplification and Sanger sequencing of the Internal Transcribed Spacer region using universal fungal primers ITS1 and ITS4 [22] on colony DNA. The resulting sequences were compared to reference data available at the Ribosomal Database Project and National Center for Biotechnology Information Genebank databases (Table 3). To confirm the presence of Saccharomyces cerevisiae subsp. boulardii, we also performed species-specific PCR for S. cerevisiae on total DNA extracted from products with primers SC1 and SC2.

**Table 3:** ITS genomic analysis ITS: Internal Transcribed Spacer; NCBI: National Center for Biotechnology Information.

	Ribosomal Database Project		NCBI Genebank	
Product	Closest organism	Score	Closest organism	% Identity
ECONORM	Saccharomyces cerevisiae	0.903	Saccharomyces sp. 'boulardii'	99.74
Gnorm	Saccharomyces cerevisiae	0.916	Saccharomyces sp. 'boulardii'	100
Reflora Z	Saccharomyces cerevisiae	0.916	Saccharomyces sp. 'boulardii'	100
Pre Pro Kid L	Saccharomyces cerevisiae	0.907	Saccharomyces sp. 'boulardii'	100
Darolac	Saccharomyces cerevisiae	0.907	Saccharomyces sp. 'boulardii'	100
Pre Pro Kid	Saccharomyces cerevisiae	0.907	Saccharomyces sp. 'boulardii'	100

## Results

We found very poor correlation between the label claim of the manufacturer and the actual contents of the probiotic samples. Because there were limitations to our analysis, such as lack of molecular analysis support, we collaborated with Universita de Cattolica, Rome, to analyze the samples using next-generation sequencing (NGS).

Viable plate count

According to the Italian Ministry of Health guidelines on probiotics and prebiotics, the number of cells present in a probiotic product must be listed on the label. Moreover, this amount has to be guaranteed until the end of the product shelf-life at the specified storage conditions, with an uncertainty of 0.5 log. To validate the label claim of CFUs, an acceptable variability of a 0.5 log factor was adopted, meaning that the product maintains the claim within a reduction of five times of the declared amount.

While checking for viable cell count, we also noted cell counts showing various colony morphologies that were not mentioned in the pack by the manufacturer. Two different colony morphologies were observed on Brain Heart Infusion plates for Benegut® (Abbott India Ltd, Mumbai, India). Because only one bacterial species is mentioned on the label content, this suggests the presence of a possible contaminant in the product.

Among the total tested probiotic products, REMUNE AL® (Sundyota Numandis Pharmaceuticals Pvt. Ltd., Ahmedabad, India) was the only product found with no viable bacterial content listed on the label. Analogously, no amplification product was obtained after PCR amplification with universal 16S rRNA gene primers, suggesting very low or nearly absent bacterial DNA content in the product. Sundyota Numandis Pharmaceuticals reports on the official website for this product that it is “[also] the first and only ‘Heat Killed’ probiotic in India” [26]. A scanning electron microscope analysis of the product found no evidence of bacterial cells in the powder.

Targeted metagenomics analysis

The probiotic products were subjected to targeted metagenomics sequence analysis to validate the bacterial composition of the label claim and to detect possible contamination. The taxonomical composition of all probiotic products was investigated through 16S rRNA-based profiling. As stated above, no amplification product was obtained for Remune AL®; the same negative result was observed for Econorm® (Dr. Reddy's Laboratories Ltd., Hyderabad, India) and Gnorm® (Nouveau Medicament, Siruseri, India) (i.e., the two food supplements based on Saccharomyces boulardii), suggesting no bacterial DNA contamination in either product. As a result, 17 samples were submitted to sequencing. Illumina-mediated 16S rRNA microbial profiling produced a total of 1,218,695 sequencing reads with an average of filtered reads of 71,688 per sample. Ninety percent (90%) were annotated at the species level; OTU counts were summarized at the species level and illustrated in Table 4. No standard protocol or recommended method exists to account for measurement errors during sample preparation and sequencing. Many studies in recent years have addressed Illumina sequencing errors by applying a global frequency threshold (typically 1%) below which variants are excluded as they are indistinguishable from quenching errors. We decided to use the 1% cut-off value.

**Table 4:** Cell count in various probiotic samples CFU: Colony-forming units; NA: Not applicable; MRS: de Man, Rogosa, and Sharpe broth.

Product	Analyzed Sample	Lot No.	Probiotic Organism(s)	Label Concentrations	Plate Count results	Warnings
Benegut	2 vials (5 ml each)	VBD0080	Bacillus clausii	2 x 10^9 ^spores/5 ml (each vial)	6.5 x 10^7 ^CFU/Vial	Two different colony morphologies have been detected on medium plates: reported count is the sum of the two
BIFILAC GG	2 sachets (0.75 g each)	AF19006	Lactobacillus rhamnosus GG	>6 x 10^9^ CFU/sachet	1.4 x 10^10^ CFU/sachet	NA
ECONORM	3 sachets (0.75 g each)	3168	Saccharomyces boulardii	250 mg/sachet	3 x 10^9 ^CFU/sachet	mg no CFUs
Cyfolac	2 vials (5 ml each)	K0118	Bacillus clausii	2 x 10^9^ spores/5 ml (each vial)	1.3 x 10^9^ CFU/vial	NA
Enterogemina	3 vials (5 ml each)	11187	Bacillus clausii	2 x 10^9 ^spores/5 ml (each vial)	1.2 x 10^9^ CFU/vial	NA
Gnorm	3 sachets (0.765 g each)	NGS 1923	Saccharomyces boulardii	250 mg/sachet	1.4 x 10^9^ CFU/ sachet	mg no CFUs
GUT PRO	2 vials (20 ml each)	XGW8002	Bacillus subtilis	>2 x 10^9^ CFU/5 ml	5.5 x 10^8^ CFU/5 ml	NA
Regutol	2 vials (30 ml each)	EP8744003	a) Bacillus subtilis, (b) Bacillus coagulan	2 x 10^9^ CFU/5 ml, 1 x 10^9^ CFU/5 ml	(a+b) 1.9 x 10^9^ CFU/5 ml	Reported count is the sum of the two organisms
SPORLAC	3 sachets (1 g each)	SPS9B013	Lactic acid Bacillus	1.5 x 10^8 ^spores/sachet	1.4 x 10^3^ CFU/sachet	NA
SuperFlora GG	2 sachets (1 g each)	12SSG015	Lactobacillus rhamnosus GG	>6 x 10^9 ^CFU/sachet	3.2 x 10^8^ CFU/sachet	NA
Vizylac	3 capsules (0.3 g each)	8065631-9092	Lactic acid Bacillus	>1.2 x 10^8 ^spores/capsule	1.7 x 10^6^ CFU/capsule	NA
Reflora Z	2 sachets (1 g each)	12SR0010	(a) Lactic acid bacillus, (b) Saccharomyces boulardii	(a) 1.5 x 10^8^ spores/sachet, (b) 2.5 x 10^9^ CFU/sachet	a) 3 x 10^7^ CFU/sachet, b) 3 x 10^8^ CFU/sachet	Counts here reported refer to results obtained with two different media according to the genus
Pre Pro kid L	3 sachets (1 g each)	FS18002	Lacto bacillus rhamnosus GG, (b) Saccharomyces boulardii	(a) 2 x 10^9 ^CFU/sachet, (b) 5 x 10^8 ^CFU/sachet	a) 3.6 x 10^7^ CFU/sachet, b) 1.6 x 10^4 ^CFU/sachet	Counts here reported refer to results obtained with two different media according to the genus
Remune AL	2 sachets (1 g each)	12SRL020	a) Lactobacillus paracasei, b) Lactobacillus fermentum	4 x 10^8 ^CFU/g	a)+b) <100 CFU/sachet	MRS medium from two different suppliers have been tested, showing the same results
Remune AL	2 sachets (1 g each)	12SRL020	a) Lactobacillus paracasei, b) Lactobacillus fermentum	4 x 10^8 ^CFU/g	a)+b) <100 CFU/sachet	MRS medium from two different suppliers have been tested, showing the same results
Darolac	3 sachets (1 g each)	M1097F358	a) Lactobacillus acidophilus, b) Lactobacillus rhamnosus, c) Bifidobacterium longum, d) Saccharomyces boulardii	>1.25 x 10^9 ^CFU/sachet	a) 1 x 10^8^ CFU/sachet, b) 3.1 x 10^7^ CFU/sachet, c) 3.2 x 10^7 ^CFU/sachet, d) 5 x 10^4 ^CFU/sachet, (1.6 x 10^8 ^CFU/sachet total)	Counts here reported refer to results obtained with a range of media according to the genus/species
Pre Pro kid	3 sachets (1 g each)	15SPR132	a) Lactobacillus acidophils, b) Lactobacillus rhamnosus, c) Bifidobacterium longum, d) Bifidobacterium infantis, e) Saccharomyces boulardii	a) 6.5 x 10^8^ CFU/sachet, b) 4 x 10^8 ^CFU/sachet, c) 1 x 10^8 ^CFU/sachet, d) 1 x 10^8 ^CFU/sachet, e) 5 x 10^7^ CFU/sachet	a) <100 CFU/sachet, b) 1.6 x 10^4^ CFU/sachet, c)+d) <100 CFU/sachet, e) 3.2 x 10^3 ^CFU/sachet	Counts here reported refer to results obtained with a range of media according to the genus/species
Combiflora	3 sachets (1 g each)	PSB18SA29	a) Lactobacillus acidophils, b) Bifidobacterium longum, c) Bifidobacterium lactis, d) Saccharomyces boulardii, e) Lactic acid Bacillus	3 x 10^9^ CFU/sachet	a) 5.8 x 10^7 ^CFU/sachet, b)+c) <100 CFU/sachet, d) <100 CFU/sachet, e) 5.2 x 10^5^ CFU/sachet, (5.8 x 10^7 ^CFU/sachet total)	Counts here reported refer to results obtained with a range of media according to the genus
Entero Plus	1 sachets (1 g each)	4797	Lactobacillus rhamnosus GG	>3 x 10^9 ^CFU/sachet	1.5 x 10^10 ^CFU/sachet	NA
BIFILAC	3 sachets (0.5 g each)	LLA9U2	a) Streptococcus faecalis, b) Clostridium butyricum, c) Bacillus mesentericus, d) Lactic acid Bacillus	a) 3 x 10^7^ CFU/sachet, b) 2 x 10^6 ^CFU/sachet, c) 1 x 10^6 ^CFU/sachet, d) 5 x 10^7^ CFU/sachet	a) 1.6 x 10^8 ^CFU/sachet, b) 3.6 x 10^7 ^CFU/sachet, c)+ d) 4.7 x 10^6^ CFU/sachet	Counts here reported refer to results obtained with a range of media according to the genus
Vibact	3 capsules (0.2 g each)	AA18D2	a) Streptococcus faecalis, b) Clostridium butyricum, c) Bacillus mesentericus, d) Lactic acid Bacillus	a) 3 x 10^7^ CFU/capsule, b) 2 x 10^6^ CFU/capsule, c) 1 x 10^6 ^CFU/capsule, d) 5 x 10^7^ CFU/capsule	a) 1.3 x 10^8^ CFU/capsule, b) 3.7 x 10^7 ^CFU/capsule, c)+d) 7 x 10^5^ CFU/capsule	Counts here reported refer to results obtained with a range of media according to the genus

The detection of Clostridium butyricum and Lactobacillus acidophilus at a low relative abundance in all products under analysis may indicate limited efficacy of the universal 16S rRNA gene primers used in this study to specifically amplify the C. butyricum and L. acidophilus V3-V4 region.

Identification of S. cerevisiae subsp. boulardi

The results of ITS sequence analysis from colony DNA are listed in Table 4. ITS sequence analysis confirmed the identity of all cultures as S. cerevisiae. Using species-specific PCR for all probiotic supplements reporting S. cerevisiae subsp. boulardi as an ingredient, the amplification product (1170 bp) specific for S. cerevisiae could be obtained. These results confirm the presence of S. cerevisiae in all tested products, including Combiflora, for which no yeast growth was obtained on yeast extract peptone dextrose (YPD) plates, thus suggesting that yeast cells lost their viability during the processing of this product.

## Discussion

The cell counts of the probiotic samples may show variation due to inherent variables in handling and storage. India is a very large country with a wide temperature gradient, and “room temperature” storage is open to a broad spectrum of bias and variability based on location. Several bacteria found in the study, such as B. cereus, were known illness-causing pathogens in humans. Other such bacteria found were Enterococcus faecium, Enterobacter cloacae, Bacillus coagulans, Pediococcus pentaseus, Bacillus subtilis, and an unidentified Gram-negative bacillus. Our study correlates very well with several studies globally [7,27,28], proving that safety and surveillance are critical during the manufacturing process. Each product in our study was analyzed using multiple techniques to eliminate any errors (e.g., colony plate count, genetic identification, phenotypic quantification, and NGS). Likewise, manufacturers should use multiple techniques to assess their products to minimize the risk of undervaluing certain microorganisms.

This is the first study of its kind in India and Asia, and the non-availability of analytical methods employed in this study may be why there are no similar studies in this region. We found no correlation between the manufacturers’ claims and the laboratory results in multiple areas such as viable cell count and genus and species contents.

## Conclusions

The goal of this study was to assess the commercial probiotic bacterial contents and their label accuracy in India, as commercial probiotics are sold as “pharmaceuticals” in India, yet no previous research has been conducted to assess probiotic quality in this population. Therefore, we conducted this Critical Analysis of Commercial Probiotics (CAMP) study. The findings from the CAMP study inform the prescribing clinician of the serious safety issues given the lack of data and quality control analysis of commercial probiotics. Therefore, regular surveillance is paramount in patient safety to optimize health outcomes for Indian patients, especially children.

## References

[1] Hill C, Guarner F, Reid G (2014). The International Scientific Association for Probiotics and Prebiotics consensus statement on the scope and appropriate use of the term probiotic. Nat Rev Gastroenterol Hepatol.

[2] Elshaghabee FMF, Rokana N, Gulhane RD, Sharma C, Panwar H (2017). Bacillus as potential probiotics: status, concerns, and future perspectives. Front Microbiol.

[3] Alfaleh K, Bassler D (2008). Probiotics for prevention of necrotizing enterocolitis in preterm infants. Cochrane Database Syst Rev.

[4] McFarland LV (2007). Meta-analysis of probiotics for the prevention of traveler’s diarrhea. Travel Med Infect Dis.

[5] Kolaček S, Hojsak I, Berni Canani R (2017). Commercial probiotic products: a call for improved quality control. A position paper by the ESPGHAN working group for probiotics and prebiotics. J Pediatr Gastroenterol Nutr.

[6] (2020). World Gastroenterology Organisation global guidelines. Probiotics and prebiotics. https://www.worldgastroenterology.org/guidelines/global-guidelines/probiotics-and-prebiotics/probiotics-and-prebiotics-english.

[7] Canganella F, Paganini S, Ovidi M, Vettraino AM, Bevilacqua L, Massa S, Trovatelli LD (1997). A microbiology investigation on probiotic pharmaceutical products used for human health. Microbiol Res.

[8] Coeuret V, Gueguen M, Vernoux JP (2004). Numbers and strains of lactobacilli in some probiotic products. Int J Food Microbiol.

[9] Drisko J, Bischoff B, Giles C, Adelson M, Rao RV, McCallum R (2005). Evaluation of five probiotic products for label claims by DNA extraction and polymerase chain reaction analysis. Dig Dis Sci.

[10] Brink M, Senekal M, Dicks LM (2005). Market and product assessment of probiotic/prebiotic-containing functional foods and supplements manufactured in South Africa. S Afr Med J.

[11] Elliot E, Teversham K (2004). An evaluation of nine probiotics available in South Africa, August 2003. S Afr Med J.

[12] Hoffman FA, Heimbach JT, Sanders ME, Hibberd PL (2008). Executive summary: scientific and regulatory challenges of development of probiotics as foods and drugs. Clin Infect Dis.

[13] (2020). Evidence for safety and efficacy of finished natural health products. http://fitomedicina.org/old/archivos/canada___legislacion_productos_naturales.pdf.

[14] Australian Government (2020). Australian regulatory guidelines for complementary medicines (ARGCM). https://www.tga.gov.au/publication/australian-regulatory-guidelines-complementary-medicines-argcm.

[15] Ganguly NK, Bhattacharya SK, Sesikeran B (2011). ICMR-DBT guidelines for evaluation of probiotics in food. Indian J Med Res.

[16] Marcobal A, Underwood MA, Mills DA (2008). Rapid determination of the bacterial composition of commercial probiotic products by terminal restriction fragment length polymorphism analysis. J Pediatr Gastroenterol Nutr.

[17] Sul SY, Kim HJ, Kim TW, Kim HY (2007). Rapid identification of Lactobacillus and Bifidobacterium in probiotic products using multiplex PCR. J Microbiol Biotechnol.

[18] Fasoli S, Marzotto M, Rizzotti L, Rossi F, Dellaglio F, Torriani S (2003). Bacterial composition of commercial probiotic products as evaluated by PCR-DGGE analysis. Int J Food Microbiol.

[19] Theunissen J, Britz TJ, Torriani S, Witthuhn RC (2005). Identification of probiotic microorganisms in South African products using PCR-based DGGE analysis. Int J Food Microbiol.

[20] Won SY, Kyeong ML, Kyu JH (2016). Taxonomic identification of bacillus species using matrix-assisted laser desorption ionization time of flight mass spectrometry. Ann Clin Microbiol.

[21] Bizzini A, Durussel C, Bille J, Greub G, Prod’hom G (2010). Performance of matrix-assisted laser desorption ionization-time of flight mass spectrometry for identification of bacterial strains routinely isolated in a clinical microbiology laboratory. J Clin Microbiol.

[22] (2020). Microbiology of food and animal feeding stuffs — General requirements and guidance for microbiological examinations. https://www.iso.org/standard/36534.html.

[23] Aureli P, Fiore A, Scalfaro C, Casale M, Franciosa G (2010). National survey outcomes on commercial probiotic food supplements in Italy. Int J Food Microbiol.

[24] Patrone V, Minuti A, Lizier M (2018). Differential effects of coconut versus soy oil on gut microbiota composition and predicted metabolic function in adult mice. BMC Genomics.

[25] Aronesty E (2013). Comparison of sequencing utility programs. Open Bioinformatics J.

[26] Sundyota Numandis Pharmaceuticals. (2019 (2020). SuperFlora™ GG sachets. https://www.sundyotanumandis.com/g-i-health.html.

[27] White TJ, Bruns T, Lee SJ, Taylor J (1989). Amplification and direct sequencing of fungal ribosomal RNA genes for phylogenetics. PCR Protocols: A Guide to Methods and Applications.

[28] Toscano M, De Grandi R, Pastorelli L, Vecchi M, Drago L (2017). A consumer’s guide for probiotics: 10 golden rules for a correct use. Dig Liver Dis.

